# Supplementary material for: Biomarkers representing key aging-related biological pathways are associated with subclinical atherosclerosis and all-cause mortality: The Framingham Study
Source: PLoS One. 2021 May 14;16(5):e0251308. doi: 10.1371/journal.pone.0251308 (PMC8121535; doi:10.1371/journal.pone.0251308)
Supplement: S1 Table — (DOCX) [file pone.0251308.s001.docx]

**S1 Table** Joint association of biomarkers of aging with incident CVD (including all 4 biomarkers)

| **Biomarker** | **HR**  **(95% CI)** | **p-value** |
| --- | --- | --- |
| IGF-1 (ng/ml) | 0.89 (0.77-1.03) | 0.12 |
| ADMA (umol/L) | 1.16 (1.02-1.32) | 0.03 |
| Isoprostane (ng/mmol) | 1.18 (1.1-1.27) | <0.0001 |
| LTL (Kb) | 0.85 (0.74- 0.99) | 0.03 |

Model adjusts for age, sex, BMI, SBP, hypertension medication, diabetes, current smoking status, total cholesterol/HDL, and eGFR.

Hazard ratios are per 1 standard deviation increase in the biomarker

Note: all four biomarkers were included in one model

Sample size: n = 801, 160 CVD events
