# Supplementary material for: Biomarkers representing key aging-related biological pathways are associated with subclinical atherosclerosis and all-cause mortality: The Framingham Study
Source: PLoS One. 2021 May 14;16(5):e0251308. doi: 10.1371/journal.pone.0251308 (PMC8121535; doi:10.1371/journal.pone.0251308)
Supplement: Attachment — Submitted filename: Response to Reviewers.docx [file pone.0251308.s002.docx]

**Response to Reviewers**

Reviewer #1:

**1. The authors mentioned (line 38) 1244 participants had available LTL measurements, but in figure 1, it showed 1386 LTL measurements were missing. It is also not clear on figure 1 if the 928 participants had the information of CAC and IMT.**

**Response:** We thank the Reviewer for this comment. We now clarify in the revised version of the manuscript that among the participants from sample 1, 928 participants had available LTL measurement (Sample 4).”, as follows:

Page 5, lines 38-39: “Finally, among the participants from **Sample 1**, 928 participants had available LTL measurements (Sample 4)”

**2. Please make statistical comparisons of biomarkers, CAC and IMT between men and women in table 1.**

**Response:** We thank the Reviewer for this suggestion. We have now performed statistical comparisons between men and women for all variable in Table 1 and included a footnote denoting significant results in the revised Table 1.

**3. On line 249, Please add references for the sentence “our finding that LTL associated with IMT is consistent with most published report.”**

**Response:** We regret this oversight. We have now cited two references, as follows:

Page 17, line 251: “Our finding that LTL is associated with IMT is consistent with most published reports. (26, 28)”
