# Supplementary material for: Biomarkers representing key aging-related biological pathways are associated with subclinical atherosclerosis and all-cause mortality: The Framingham Study
Source: PLoS One. 2021 May 14;16(5):e0251308. doi: 10.1371/journal.pone.0251308 (PMC8121535; doi:10.1371/journal.pone.0251308)
Supplement: Attachment — Submitted filename: Response to Reviewers Final.docx [file pone.0251308.s003.docx]

**Response to Reviewers**

PONE-D-20-32445R1
Biomarkers representing key aging-related biological pathways are associated with subclinical atherosclerosis and all-cause mortality: The Framingham Study

**Reviewer #2: In the current article, authors prospectively investigated the associations between aging related biomarkers with subclinical atherosclerosis and all-cause mortality. However, there are some issues need to be noted:**

**1. Do the 928 patients used for LTL analyses have both IMT and CAC data? Please report the number of deaths in LTL group.**

**Response:** We thank the Reviewer for this question. We would like to clarify that not all 928 participants included in analyses had available data on both IMT and CAC. Among the 928 participants, 670 had data on IMT, 253 had data on CAC, and 223 participants had data on both IMT and CAC. In addition, there were 250 deaths among the 928 participants used for LTL analyses. We have now added this information in the revised version of the manuscript, as follows:

Page 5, lines 63-65: “Of note, among the 928 participants with LTL measurements available, 670 had data on IMT, 253 had data on CAC, and 223 participants had data on both IMT and CAC. IMT and CAC values were measured at examination 8 (2005-2008) and 2002-2005, respectively.”

**2. IMT and CAC were measured at the eighth follow-up visit, and were not performed simultaneously with the sixth and seventh blood draws and urine retention. The time interval may be more than eight years apart, so they are not cross-sectional analyses.**

**Response:** We thank the Reviewer for this question. It is correct that not all measures were performed simultaneously and that the time interval between collection of some of the biomarkers and IMT and CAC is more than eight years. ADMA and LTL were assessed at examination cycle 6 (1995-1998), IMT at examination cycle 8 (2005-2008), and CAC during 2002-2005. Since we are not following participants for incidence of events, we have defined this analysis as a cross-sectional investigation. However, if the Reviewer feels strongly, we are happy to remove all mention of “cross-sectional” from the text. We have also added the following in the revised version of the manuscript:

Page 5, lines 64-65: “IMT and CAC values were measured at examination 8 (2005-2008) and 2002-2005 for CAC, respectively.”

**3. The results of univariate analyses without adjusting confounding factors need to be reported.**

**Response:** To address the Reviewer’s concern, we have now updated **Table 2**, in which we have included the estimates (and 95% CI) for unadjusted models relating aging biomarkers with IMT and CAC.

**Revised Table 2. Association of individual biomarkers of aging with components of subclinical atherosclerosis.**

| **Biomarker*** | **Unadjusted model** |  | **Model 1** |  | **Model 2** |  |
| --- | --- | --- | --- | --- | --- | --- |
| **Association with CAC^†^** | **Estimate**  **(95% CI)** | **p-value** | **Estimate**  **(95% CI)** | **p-value** | **Estimate**  **(95% CI)** | **p-value** |
| LTL (Kb) | -0.63 (-0.95, -0.31) | 0.0002 | -0.22 (-0.5, 0.06) | 0.12 | -0.24 (-0.51, 0.04) | 0.09 |
| IGF-1 (ng/ml) | -0.07 (-0.25, 0.10) | 0.40 | 0.16 (0.0, 0.32) | 0.05 | 0.03 (-0.12, 0.17) | 0.71 |
| ADMA (umol/L) | 0.48 (0.30, 0.65) | <.0001 | -0.01 (-0.15, 0.13) | 0.91 | **0.25 (0.11, 0.39)** | **0.001** |
| F2-Isoprostane (ng/mmol) | -0.10 (-0.29, 0.10) | 0.33 | 0.25 (0.11, 0.40) | 0.0006 | 0.06 (-0.10, 0.22) | 0.47 |
| **Association with IMT^‡^** |  |  |  |  |  |  |
| LTL (Kb) | -0.22 (-0.29, -0.16) | <.0001 | -0.09 (-0.15,-0.03) | 0.01 | **-0.08 (-0.14, -0.02)** | **0.01** |
| IGF-1 (ng/ml) | -0.08 (-0.12, -0.04) | <.0001 | 0.06 (0.03,0.1) | <0.0001 | **-0.04 (-0.08, -0.01)** | **0.02** |
| ADMA (umol/L) | 0.10 (0.06, 0.14) | <.0001 | -0.06 (-0.1,-0.02) | <0.0001 | 0.02 (-0.02, 0.05) | 0.34 |
| Isoprostane (ng/mmol) | 0.02 (-0.02, 0.07) | 0.27 | 0.03 (-0.01,0.07) | 0.12 | 0.02 (-0.02, 0.06) | 0.24 |

*Biomarkers were analyzed in separate models

**^†^** CAC was modeled as ln(CAC+1)

**^‡^** IMT was modeled as mean of standardized CCA IMT and ICA IMT

Model 1 is adjusted for age and sex.

Model 2 is adjusted for age, sex, BMI, SBP, hypertension medication, diabetes, current smoking status, and total cholesterol/HDL

Estimates are per 1 standard deviation increase in the biomarker

Sample sizes: n_CAC_ = 890	n_IMT_ = 1697	n_LTL_ = 928

**4. Table 1 compares the characteristics of the population between sex, so whether the relationships between biomarkers and outcomes are different in male and female?**

**Response:** We thank the Reviewer for raising this important question. To address the Reviewer’s comment, we evaluated potential effect modifications of the relation between aging biomarkers and death by sex and display results in **Reviewer Table 1** below. We did not observe any significant effect modifications by sex. We also added the following in the revised version of the manuscript:

Page 10, Line 156-159: “We also evaluated potential effect modification of the relation between biomarkers of aging and components of subclinical atherosclerosis by sex by including corresponding interaction terms in the models. A P-value <0.05 for the interaction was considered statistically significant.”

Page 14, Line 220-221: “We did not observe significant effect modifications of the relation between biomarkers of aging and subclinical disease indices by sex (all p values exceeded 0.15)”

**Reviewer Table 1. Evaluation of effect modification of the relation between biomarkers of aging and subclinical disease indices by sex.**

| **Biomarker*** | **Estimate**  **(95% CI)** | **p-value** |
| --- | --- | --- |
| **Association with CAC^†^** |  |  |
| LTL*Male | -0.38(-0.93,0.17) | 0.17 |
| IGF-1*male | 0.05(-0.24,0.34) | 0.73 |
| ADMA*male | 0.09(-0.19,0.37) | 0.52 |
| F2-Isoprostane*male | 0.04(-0.32,0.39) | 0.84 |
| **Association with IMT^‡^** |  |  |
| LTL*male | 0.01(-0.11,0.13) | 0.82 |
| IGF-1*male | -0.04(-0.11,0.03) | 0.29 |
| ADMA*male | -0.01(-0.08,0.06) | 0.72 |
| F2-Isoprostane*male | -0.05(-0.12,0.02) | 0.19 |

**5. In the analysis of individual biomarkers and mortality risk, each marker has predictive value. So, just because the number of patients with LTL data is small, then taken this parameter out of the subsequent analysis model, and calculated the biomarker score using the other three parameters, is not rigorous.**

**Response:** We thank the Reviewer for raising this important point. To address the Reviewer’s comment, we have now created a score that includes all four biomarkers and have added results in Figure 2b and in the text, as follows:

Page 11, lines 184-186: “In secondary analysis we created a biomarker score including all four biomarkers (IGF-1, ADMA, F2-Isoprostanes, and LTL).”

**6. Please clarify the age range of the study population.**

**Response:** We thank the Reviewer for this comment. We have now added the age range of the study sample as follows:

Page 12, line 198: “…with an age range of 33 to 88 years,…”

**7. In the discussion section, the authors repeatedly mentioned the influence of the age of the study population on the research results. So according to the author's understanding, which age group has the most predictive value of the relationship between these biomarkers and outcome events?**

**Response:** We thank the Reviewer for raising this point. The focus of our investigation was to evaluate the relation of aging biomarkers with indices of subclinical disease and all-cause mortality. We did not focus on performing prediction models in this investigation. We hope the Reviewer agrees with our approach.
