# Supplementary material for: Biomarkers representing key aging-related biological pathways are associated with subclinical atherosclerosis and all-cause mortality: The Framingham Study
Source: PLoS One. 2021 May 14;16(5):e0251308. doi: 10.1371/journal.pone.0251308 (PMC8121535; doi:10.1371/journal.pone.0251308)
Supplement: Attachment — Submitted filename: Response to Reviewers_April 2021.docx [file pone.0251308.s004.docx]

**Response to Reviewers**

PONE-D-20-32445R2
Biomarkers representing key aging-related biological pathways are associated with subclinical atherosclerosis and all-cause mortality: The Framingham Study

**Reviewer #2:**

1. **In Table 2, since the sample size differed from each biomarker, especially for LTL, the study sample size should be labeled after the biomarkers.**

**Response:** We thank the Reviewer for this point. We have now added the sample size after each biomarker in Table 2.

**2. When considered LTL in the multivariate model, the sample size of Table S1 should	be 2010.**

**Response:** We thank the Reviewer for this comment, we have now revised Table S1 to address the comment.
